# Synthesis of a Cyclophosphazene Derivative Containing Multiple Cyano Groups for Electron-Beam Irradiated Flame-Retardant Materials

**DOI:** 10.3390/polym13203460

**Published:** 2021-10-09

**Authors:** Bingbing Leng, Jiayu Yang, Chunhui Zhu, Zhipeng Wang, Chengying Shi, Yang Liu, Hongyan Zhang, Wenge Xu, Baijun Liu

**Affiliations:** 1 Changchun Power Supply Company, State Grid (Jilin Province) Electric Power Co., Ltd., 4969 Renmin Street, Changchun 130021, China; lengbingbingmail@sina.com (B.L.); zhipengwang162@163.com (Z.W.); 2 National & Local Joint Engineering Laboratory for Synthesis Technology of High Performance Polymer, College of Chemistry, Jilin University, 2699 Qianjin Street, Changchun 130012, China; jiayuy20@mails.jlu.edu.cn (J.Y.); sicangying@foxmail.com (C.S.); 3 Economic and Technological Research Institute, State Grid (Jilin Province) Electric Power Co., Ltd., 1427 Pingquan Road, Changchun 130062, China; chunhuizhu163@163.com; 4 Changchun Radiation Technology Co., Ltd., China Isotope & Radiation Corporation (CIRC), 770 Shengye Road, Changchun 130102, China; liuyang@circ.com.cn (Y.L.); zhanghongyan@circ.com.cn (H.Z.); xuwenge@circ.com.cn (W.X.)

**Keywords:** EB-irradiation, cyclophosphazene derivative, crosslinked networks, flame retardancy, wires and cables

## Abstract

A cyclophosphazene derivative containing multiple cyano groups, denoted as hexa(4-cyanophenoxy)cyclotriphosphazene (CN-CP), was synthesized by a one-step nucleophilic substitution reaction for a phosphorus-nitrogen flame retardant. To meet the strict requirement of safe and environment-protective insulation materials, a series of composites based on low-density polyethylene-poly(ethylene-co-vinyl acetate) containing CN-CP/Mg(OH)_2_/Al(OH)_3_ organic-inorganic synergistic flame retardants was fabricated. High-energy electron beam irradiation was subsequently applied to obtain a halogen-free flame-retardant crosslinked system. The relationship between crosslinking degree and irradiation dose was studied, and crosslinking degrees ranging within 63–85% were obtained under 100–190 kGy. Furthermore, the effects of CN-CP filler and irradiation dose on the properties of the composites were carefully investigated. The maximum tensile stress and limiting oxygen index values of most composites irradiated by EB were more than 15 MPa and 28%. Results revealed that the obtained materials had excellent thermal and mechanical, flame-retardant, and insulation properties, thereby suggesting their promising prospects for wire and cable applications.

## 1. Introduction

Low-density polyethylene (LDPE) is one of the most widely used plastics. It is known for its low cost, easy processability, and excellent mechanical and insulating properties [1]. With the continuous increase in public environmental awareness, nontoxic and halogen-free LDPE is believed to a promising candidate to replace traditional polyvinylchloride as insulation layers in wires and cables [2]. To meet performance requirements, poly(ethylene-co-vinyl acetate) (EVA) elastomer is often introduced into an LDPE matrix to obtain LDPE/EVA blends with improved flexibility, toughness, and resistance to environmental stress cracking of insulations [3,4]. Unfortunately, the flammable nature of polyolefins limits their extensive application as wire and cable insulation.

The flame retardancy of polyolefins can be enhanced by the incorporation of organic and inorganic fillers. Owing to the high-efficiency flame-retardant and smoke-suppression effects of some metallic hydroxide fillers (e.g., Mg(OH)_2_ and Al(OH)_3_ nanoparticles), they are widely applied in halogen-free flame-retardant materials [5,6]. However, several unfavorable phenomena are often observed when these inorganic fillers are introduced into the polymer matrix. To provide adequate flame-retardancy effects, an extremely high loading amount of metallic hydroxides (e.g., ≥65%) must be present in the composites [7]. The high content of inorganic particles in the polymer matrix always leads to a significant deterioration of flexibility and other properties. To improve the compatibility between the organic matrix and inorganic fillers, the fillers (e.g., Mg(OH)_2_ and Al(OH)_3_) have to be ground into nanoparticles and then treated by surfactants [8,9,10]. Therefore, the development of novel compound flame retardants is important to replace or partially replace currently used inorganic flame retardants.

Crosslinking is a critical approach to improving the thermomechanical properties and chemical resistance of LDPE-based materials. Compared with chemical crosslinking, electron-beam (EB) crosslinking has many advantages [11,12,13], including the following: (1) crosslinking occurs in the absence of any catalyst, which avoids the residue of small molecules in the products; (2) crosslinking is fast and clean, which is suitable for industrial production; (3) crosslinking degree is precisely controllable by the irradiation dosage; and (4) crosslinking reaction can be conducted at room temperature. Several investigations have indicated that the blends of LDPE and EVA are susceptible to the formation of integrated crosslinked networks under EB-irradiation [14,15]. Cyclophosphazene derivatives (CPs) are believed to be a family of highly efficient flame retardants because of their unique nitrogen/phosphorus-rich heterocyclic structure, which may exert synergistic effects on the improvement of flame-retardant performance [16]. For instance, an allyl-functionalized CP has been incorporated into poly(ethylene terephthalate) to form a flame-resistant blend with a limiting oxygen index (LOI) value of 33.5%, which was much higher than that (26.5%) of pure PET [17]. However, few CPs have been investigated for EB-irradiated flame retardant PE/EVA blends.

In the current study, a CP containing multiple cyano groups was synthesized through a fast and simple nucleophilic substitution reaction. The CP can serve as a complexed halogen-free flame retardant together with Mg(OH)_2_ and Al(OH)_3_ particles and an irradiation sensitizer in a PE/EVA blend. Its high phosphorus and nitrogen contents and large number of polar cyano groups were expected to improve flame retardancy and enhance interactions among various components in the PE/EVA system. The properties of the composites after EB-irradiation at different doses were thoroughly investigated. The crosslinked PE/EVA-based composites containing organic-inorganic complex flame retardants after EB-irradiation were confirmed to have many attractive properties for wire and cable applications.

## 2. Materials and Methods

### 2.1. Materials

LDPE was purchased from LG Chem, Ltd. (Yeoui-daero Yeongdeungpo-gu Seoul, Seoul, Korea), and its number-average molecular weight of LDPE was about 150,000–250,000. Poly(ethylene-co-vinyl acetate) (VA content: ~15%) was obtained from Shanghai PUEN Chemical Co., Ltd. (Shanghai, China). Mg(OH)_2_ and Al(OH)_3_ particles (industrial grade) were purchased from Zibo Chenyu Fine Powder Co., Ltd., (Shandong, China). Hexachlorocyclotriphosphazene (99%) was obtained from Wuhan Hezhong Bio-chemical Manufacture Co., Ltd., (Wuhan, China). 4-Cyanophenol (98%) was supplied by Shanghai Aladdin Biochemical Technology Co., Ltd., China. Potassium carbonate (K_2_CO_3_, 99%) was obtained from Sinopharm Chemical Reagent Co., Ltd., (Shanghai, China). All other chemicals and reagents were from commercial resources and used without purification.

### 2.2. Instruments and Measurements

Fourier transform infrared spectroscopy (FTIR) was carried out on a Nicolet Impact 410 spectrometer at 4000−400 cm^−1^. Proton nuclear magnetic resonance (^1^H NMR) spectroscopy was run on a Bruker 510 spectrometer (500 MHz), and CDCl_3_ was selected as a solvent. The microstructure of the membranes was observed on a Nova NanoSEM 450 scanning electron microscopy (FEI Company, Hillsboro, OR, USA). Thermogravimetric analysis (TGA) was conducted on a Perkin Elmer Pyris-1 instrument under N_2_ at a heating rate of 10 °C·min^−1^.

A SHIMADZU AG-I 1KN instrument was used to test the mechanical properties of the membranes (15 mm × 5 mm) at a strain rate of 2 mm·min^−1^.

Sheets were irradiated using an electron accelerator (Rhodotron TT200, Louvain-la-Neuve, Belgium) with 3 MeV energy under various irradiation doses (100–190 kGy).

The gel content of the crosslinked samples was tested using an extraction method, and xylene was used as solvent.

LOI measurements were carried out on an M606B oxygen-index apparatus (China). The specimen dimensions were 4 mm × 10 mm × 100 mm.

The UL-94 vertical burning level was tested on an FTT0082 instrument (Fire Testing Technology, East Grinstead, UK) according to ASTM 3801. The sample dimensions were 125 mm × 12.7 mm × 3.2 mm.

Surface and volume resistance were measured at room temperature by using a ZC-90F teraohmmeter (Shanghai Hanyi Electrical Technology Co., Ltd., Shanghai, China).

A dielectric-loss measurement system (model Automatic LCR Meter 4225; Tianji Anfutai Electrical Technology Co., Ltd., Tianjin, China) was used to determine the dielectric constant and the dielectric-loss tangent of the samples.

### 2.3. Synthesis of a Cyano-Functionalized CP

**Scheme 1:**
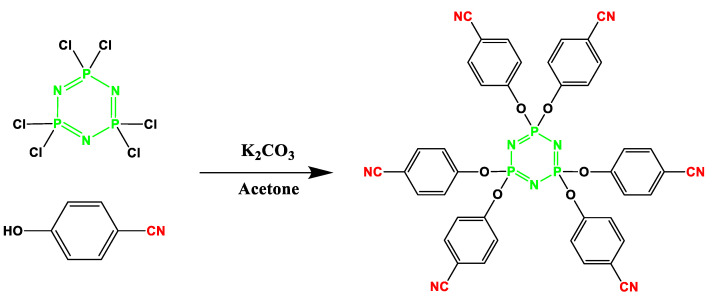
Synthesis route of CN-CP.

We added 17.2 g (0.144 mol) of 4-hydroxybenzonitrile, 21.9 g (0.16 mol) of K_2_CO_3_, and 200 mL of acetone into a 500 mL three-necked flask equipped with a condenser and a mechanical stirrer. After stirring at reflux temperature under N_2_ for 4 h, a solution of 6.953 g (0.02 mol) of hexachlorocyclotriphosphazene in 20 mL of acetone was added dropwise into the reaction mixture. The reaction was continued at 56 °C for another 6 h, and the solution was poured into deionized water. The precipitate was washed with deionized water and acetone three times, and a white powder was obtained. After drying in a vacuum oven at 80 °C for 12 h, hexa(4-cyanophenoxy)cyclotriphosphazene (CN-CP) was obtained (Scheme 1). The yield was about 83%.

### 2.4. Preparation of Insulation-Composite Materials Containing Inorganic-Organic Flame Retardants

Halogen-free flame-retardant insulation-composite materials containing LDPE, EVA, Mg(OH)_2_, Al(OH)_3_, and CN-CP were prepared using a melt-blending method at 125 °C with an open mill followed by a hot-pressing mold at 125 °C under 3.0 MPa with a plate vulcanizing machine. According to the formulation illustrated in Table 1, the samples were denoted as PE-CN-X (X = 0, 1, 2, 3, 4, and 5).

### 2.5. Preparation of EB-Irradiated Insulation Materials

The obtained sheets were irradiated with a high-energy (3.0 MeV) electron accelerator under various irradiation doses (100–190 kGy). Finally, crosslinked composites with different crosslinking degrees were obtained for further performance investigation.

## 3. Results and Discussion

### 3.1. Synthesis of Multiple Cyano-Containing CP as Organic Flame Retardant

The development of novel organic flame retardants for use in insulation materials is continuously required [18,19,20]. CPs usually have high contents of phosphorus and nitrogen elements, which may play important roles in flame retardancy [21,22]. In the current study, multiple cyano groups (–CN) were readily attached onto a cyclotriphosphazene unit under mild conditions via nucleophilic substitution (Scheme 1). The product was named CN-CP, and it possessed a unique six-arm chemical structure bearing polar –C≡N groups.

**Figure 1:**
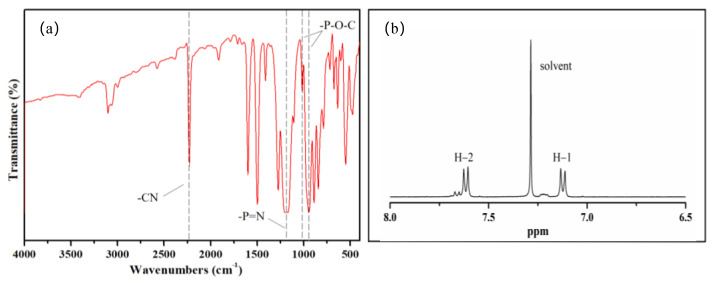
FTIR (**a**) and ^1^H NMR (**b**) spectra of CN-CP.

FTIR, ^1^H NMR, and elemental analyses were used to identify the chemical structure of CN-CP. In its FTIR spectrum (Figure 1a), 1205 cm^−1^ belonged to the characteristic absorption peak of P=N, and 939 and 1105 cm^−1^ were the characteristic absorption peaks of P–O–C. A strong absorption at 2227 cm^−1^ caused by –CN groups was clearly observed. In its ^1^H NMR spectrum (Figure 1b), doublets appeared at 7.61 and 7.12 ppm from H2 and H1 on the benzene ring of CN-CP, separate from the characteristic absorption of deuterated chloroform at 7.26 ppm. Results of elemental analysis (C = 76.674%, H = 3.956%, N = 19.370%) for CN-CP were relatively close to its theoretical values (C_42_H_8_O_6_N_9_P_3_: C = 77.051%, H = 3.695%, N = 19.255%)

### 3.2. Preparation of EB-Irradiated LDPE/EVA Composites Containing CN-CP

**Figure 2:**
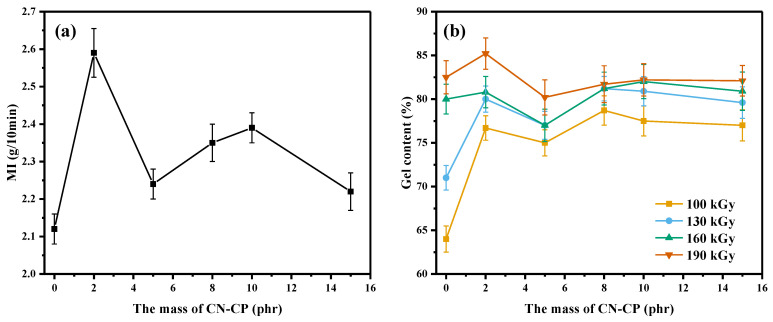
Melt index (**a**) and relationship between radiation dose and gel content (**b**) of the composites.

As is illustrated by the formulation in Table 1, halogen-free flame-retardant composites were prepared using a melting-blending process. To evaluate the effect of CN-CP on processability, the melt index of the composites with different CN-CP contents was determined. As is shown in Figure 2a, the incorporation of CN-CP slightly improved the melt index, and PE-CN-1 containing 2 g CN-CP showed the highest value of 2.6 g/10 min.

EB-irradiation was applied to yield stable, crosslinked PE/EVA networks as an efficient method of enhancing mechanical strength and heat resistance. In many cases, a high crosslinking degree above 70% is needed for PE-based materials to be used in the field of cables and tubes. In the present study, the crosslinking degree of composites was evaluated by measuring gel content after irradiation at doses of 100, 130, 160, and 190 kGy. Figure 2b shows that for each sample, a higher irradiation dose always led to a higher crosslinking degree. Under the highest irradiation dose of 190 kGy, the crosslinking degrees of all test samples exceeded 80%. Notably, the CN-CP filled composites always exhibited higher crosslinking degrees than those without CN-CP (PE-CN-0). We deduced that CN-CP with unsaturated C≡N and P=N groups may serve as an irradiation-crosslinking sensitizer and play a role in promoting the irradiation crosslinking of the composites.

**Figure 3:**
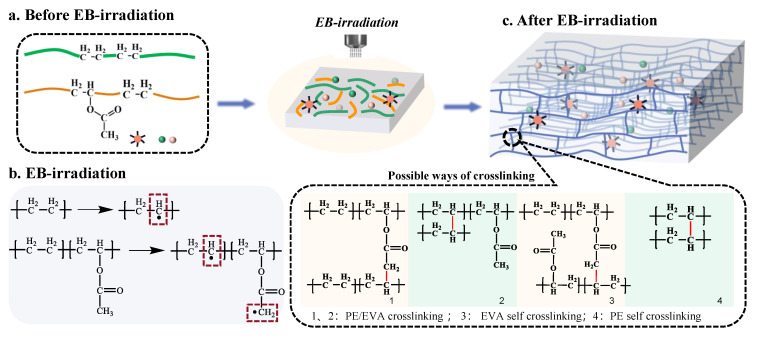
Illustration of crosslinked PE/EVA networks containing organic-inorganic flame retardants.

Diagrams of composite preparation and EB-irradiation processes are presented in Figure 3. The above analyses confirmed that upon EB-irradiation, highly crosslinked PE/EVA networks, in which organic CN-CP and inorganic Mg(OH)_2_/Al(OH)_3_ particles were distributed, were fabricated. This composition can be expected to have the desired combination of high strength, good heat resistance, sufficient flexibility, and excellent flame retardancy.

### 3.3. Morphology of Composites

**Figure 4:**
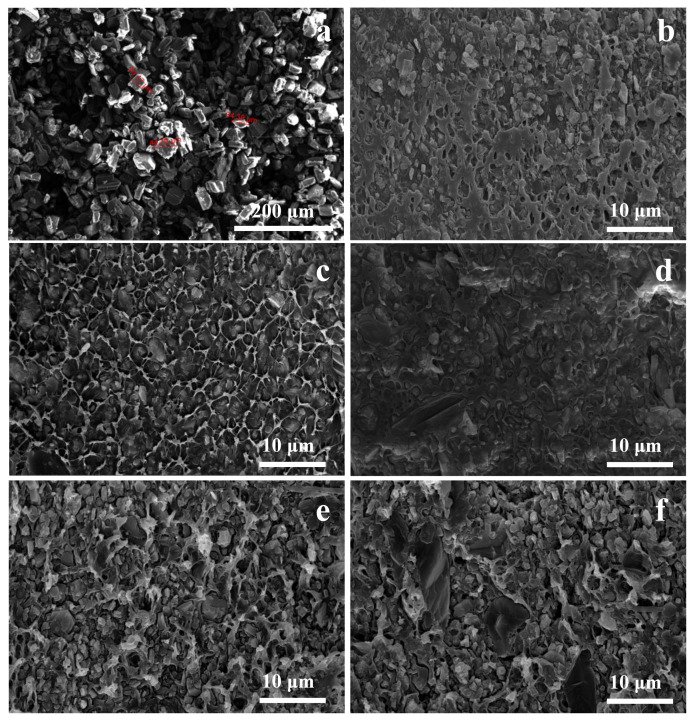
SEM images of CN-CP powder and its composites (**a**) CN-CP powder; (**b**) PE-CN-1; (**c**) PE-CN-2; (**d**) PE-CN-3; (**e**) PE-CN-4; (**f**) PE-CN-5.

The morphology of CN-CP powder and its composites was investigated by SEM; the results are shown in Figure 4. The size of CN-CP powder before blending was around 20–40 μm (Figure 4a). For all composite samples, fillers including CN-CP and inorganic Mg(OH)_2_/Al(OH)_3_ powder were evenly dispersed in the LDPE/EVA matrix (Figure 4b–f). Even at a higher load of CN-CP, no obvious aggregation of fillers was observed. This finding was due to CN-CP being an organic substance, i.e., it had good affinity for the PE/EVA matrix.

**Figure 5:**
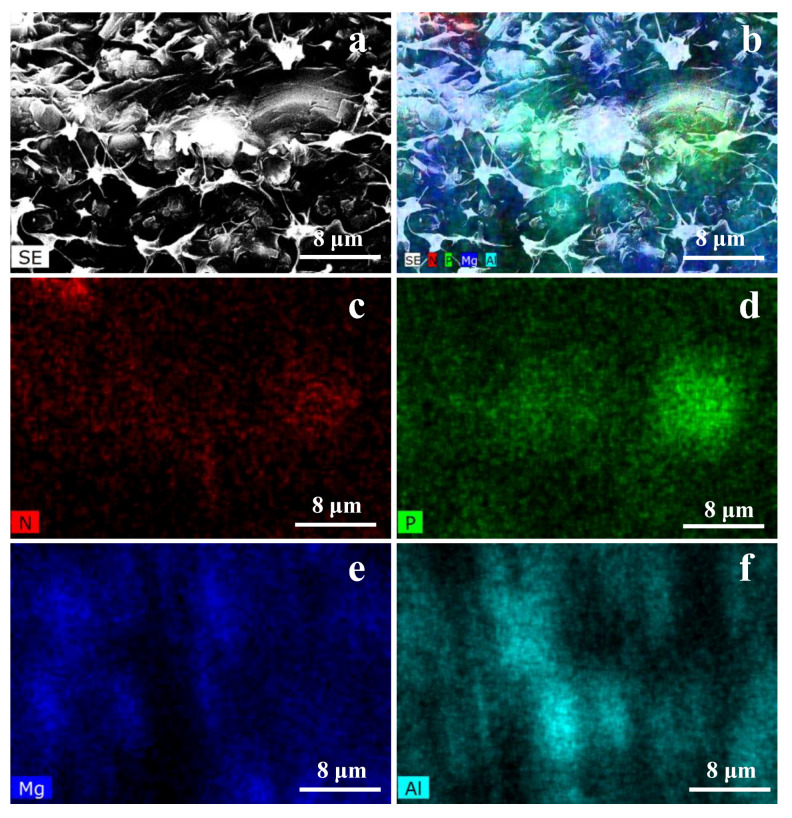
SEM (**a**) and EDS images (**b**–**f**) of PE-CN-5.

The EDX mapping images of N, P, Al, and Mg elements for the PE-CN-5 sample with a high CN-CP content are shown in Figure 5. We observed that N/P elements from organic fillers and Al/Mg elements from inorganic fillers were uniformly dispersed throughout the composites. Additionally, no Cl element was observed in the EDS spectrum, suggesting a complete substitution reaction during the preparation process of the CN-CP flame retardant.

### 3.4. Thermal and Mechanical Properties

The thermal stability of the composites was evaluated by TGA in air. We found that no thermal weight loss occurred below 200 °C, and the composites began to lose weight beyond 250 °C owing to the degradation of LDPE and EVA. CN-CP introduction and EB-irradiation treatment had no serious influence on the thermal stability of the composites. The residues at 700 °C of the crosslinked samples ranged within 20–30%, and they were believed to be caused by the Mg(OH)_2_/Al(OH)_3_ fillers.

**Figure 6:**
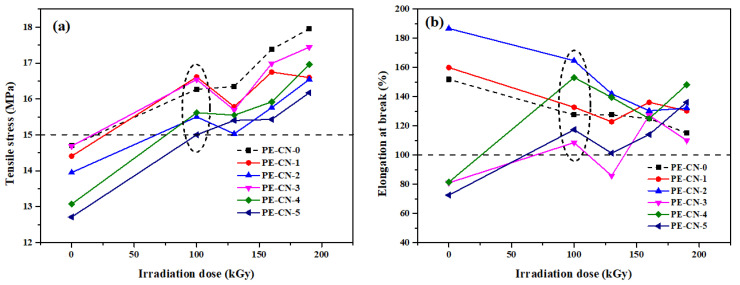
Tensile stress (**a**) and elongation at break (**b**) of the composites under different irradiation doses.

We subsequently conducted the tensile test of the composites before and after irradiation was conducted, as summarized in Figure 6. Results showed that the maximum tensile strength of the composites increased with increased irradiation dose, indicating that EB-irradiation helped improve the tensile strength of the composites. Furthermore, higher irradiation doses (>130 kGy) caused the simultaneous occurrence of crosslinking and degradation of the LDPE and EVA molecular chains, leading to a more complicated effect on tensile properties. This contradiction brought a fluctuation to the increasing trend of the strengths (Figure 6a). In some cases, the incorporation of CN-CP components even led to improved tensile properties. For example, at 100 kGy, the tensile strengths of PE-CN-1 and PE-CN-3 reached 16.61 and 16.54 MPa, which were higher than that (16.27 MPa) of PE-CN-0. PE-CN-1 and PE-CN-4 also had enhanced elongations at break compared with PE-CN-0 without CN-CP filler. Overall, the maximum tensile strength and elongation at break of most composites irradiated by high-energy EB were more than 15 MPa and 100%, respectively. These desired mechanical properties provided sufficient strength and flexibility for the composites to be used in wires and cables.

### 3.5. Flame-Retardancy and Electrical-Insulation Properties

**Figure 7:**
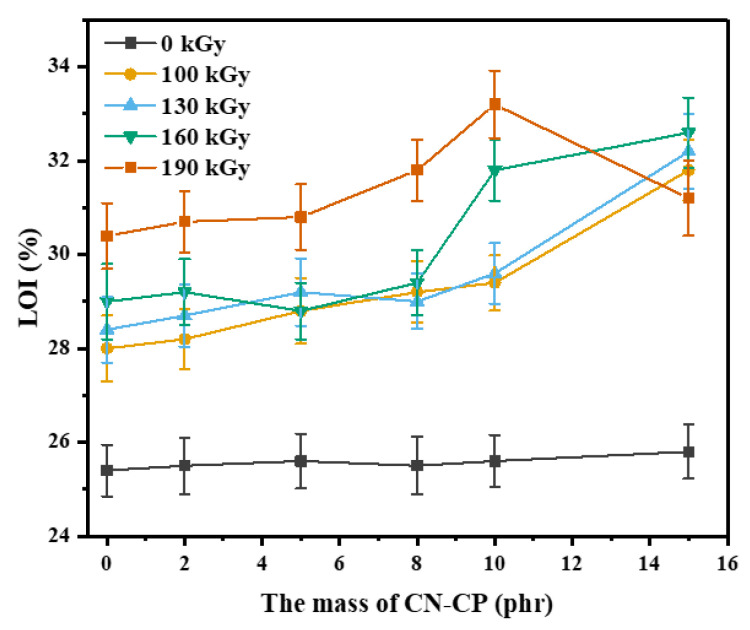
Limiting oxygen index (LOI) of the composites under different irradiation doses.

The LOI of the composites under different irradiation doses was tested to evaluate their flame retardancy (Figure 7). Flame-retardant contents and irradiation doses clearly affected flame retardancy. First, current organic-inorganic synergistic flame retardants (CN-CP/Mg(OH)_2_/Al(OH)_3_) exhibited excellent flame-retardant performance. The LOI values of PE-CN-1, 2, 3, 4, and 5 ranged within 26–33%, which was much higher than that (17.6%) of the pristine LDPE/EVA sample. Second, high-energy EB-irradiation played a significant role in increasing LOI values, and samples treated at a higher dose generally had a higher LOI value. For instance, at 190 kGy, the LOI value of PE-CN-4 was 33%, which was 26.9% higher than that of the sample without irradiation. The vertical burning levels of PE-CN-1, 2, 3, 4, and 5 containing organic-inorganic flame retardants reached V-1 and V-0, and it was confirmed that the CN-CP organic filler helped enhance the burning level (Table 2).

Resistance, volume resistivity, relative permittivity, and capacitance were measured to characterize the insulation performance of the halogen-free flame retardant composites. As is shown in Table 2, the introduction of CN-CP filler had no obvious effect on the insulation properties of the composites. Resistance and volume resistivity were above 1.9 × 10^12^ Ω and 4.0 × 10^14^ Ω·cm, respectively. Relative permittivity and capacitance were above 2.6 and 50 pF, respectively. Clearly, these properties met the requirements of wire and cable insulation materials.

## 4. Conclusions

Based on the successful synthesis of a cyano-functionalized CN, a new family of halogen-free flame-retardant insulating materials comprised of an LDPE/EVA matrix and organic-inorganic synergistic flame retardants were fabricated for wire and cable applications. High-energy EB-irradiation was used to prepare crosslinked networks and enhance the performance of the composites. The relationships among properties, composition, and irradiation dose were carefully investigated. EB-irradiated composites were found to exhibit many attractive properties, including high mechanical strength (tensile stress: ≥15 MPa), excellent flame retardancy (LOI: 25.2–33.0%), and good electrical-insulating properties (resistance: ≥1.9 × 10^12^ Ω; volume resistivity: ≥4.0 × 10^14^ Ω·cm). These combined findings suggest promising application prospects for these PE/EVA-based insulating materials.

## Figures and Tables

**Table 1:** Formulation of insulation materials.

Sample	PE (phr)	EVA (phr)	Mg(OH)_2_ (phr)	Al(OH)_3_ (phr)	HPCTP-CN (phr)
PE-CN-0	100	40	100	100	0
PE-CN-1	100	40	100	100	2
PE-CN-2	100	40	100	100	5
PE-CN-3	100	40	100	100	8
PE-CN-4	100	40	100	100	10
PE-CN-5	100	40	100	100	15

**Table 2:** UL-94 vertical burning level and insulation properties of PE-CN composites.

Sample	UL-94	Resistance(Ω)	Volume Resistivity(Ω·cm)	Capacitance(pF)	Permittivity
PE-CN-0	NR	2.75 × 10^12^	5.68 × 10^14^	55	2.8
PE-CN-1	V-1	1.90 × 10^12^	4.23 × 10^14^	56	2.6
PE-CN-2	V-0	2.70 × 10^12^	5.41 × 10^14^	54	2.8
PE-CN-3	V-0	2.81 × 10^12^	5.52 × 10^14^	53	2.8
PE-CN-4	V-0	3.22 × 10^12^	6.02 × 10^14^	50	2.8
PE-CN-5	V-0	2.00 × 10^12^	4.05 × 10^14^	55	2.8

## Data Availability

Not applicable.
